# Concordance of DLQI and pain score in hidradenitis suppurativa clinical trials: a systematic review

**DOI:** 10.1097/JW9.0000000000000204

**Published:** 2025-05-16

**Authors:** Adina Greene, Angelina S. Hwang, Sarah Amjad, Jacob A. Kechter, Aaron R. Mangold, Stella X. Chen

**Affiliations:** a University of Arizona College of Medicine, Phoenix, Arizona; b Mayo Clinic Department of Dermatology, Scottsdale, Arizona; c Mayo Clinic Alix School of Medicine, Scottsdale, Arizona

**Keywords:** biologics, DLQI, hidradenitis suppurativa, HiSCR, pain, patient-reported outcomes

## Abstract

**Background:**

Hidradenitis suppurativa (HS) is a chronic, recurrent inflammatory condition and is associated with significant psychosocial impacts on patient quality of life.

**Objective:**

This study aimed to characterize the utilization of patient-reported outcomes (PROs) in HS clinical trials and their concordance with trial primary endpoints.

**Methods:**

A systematic review of clinical trials was performed using the publicly available U.S. National Library of Medicine (ClinicalTrials.gov) in June 2023 utilizing the search terms hidradenitis, hidradenitis suppurativa, and suppurativa hidradenitis. Study title, start year, trial status, intervention, design, location, and primary and secondary outcomes were collected. To assess for concordance of patient and provider-reported outcomes, we identified published placebo-controlled trials that included Dermatology Life Quality Index (DLQI) and/or a numeric rating scale (NRS) for pain, the 2 most utilized PROs.

**Results:**

One hundred sixty-four HS clinical trials were identified, of which 115 were interventional studies. A total of 65.2% (*n* = 107) of HS trials included at least one PRO. Pain NRS (42.7%, *n* = 70) and DLQI (42.1%, *n* = 69) were the most frequently used PRO instruments. The use of PROs in clinical trials increased over time, with 50% of trials between 2020 and 2023 utilizing PROs. Of 11 published HS trials, 82% (*n* = 9) trials showed concordance of provider-assessed and PROs in at least one treatment arm.

**Limitations:**

Significance and standard deviations of PROs were rarely reported, preventing the calculation of significance and is a limitation of this review.

**Conclusion:**

While lesion counts provide single snapshots of disease, PROs can capture perceptions of lesions not actively present. PROs are frequently concordant with both achievement and nonachievement of primary endpoints in clinical trials.

**What is known about this subject with regard to women and their families?**

- Hidradenitis suppurativa (HS) is a chronic inflammatory condition most commonly affecting the apocrine gland-bearing regions and is associated with significant psychosocial impacts on patient quality of life.
- In Western countries, HS has been documented to affect a larger proportion of females compared to males.

**What is new from this article as messages for women and their families?**

- Our study demonstrates a high concordance between provider and patient-assessed outcomes across HS trials, which can capture perceptions of lesions not actively present.
- This type of study can potentially help more accurately capture the severity of HS flares and efficacy of medications with respect to the management of HS.
- As HS commonly affects females and can have great impacts on their quality of life, we hope that this study will contribute to the management of HS in females with a focus on patient-centered care.

## Introduction

Patient-reported outcomes (PROs) in clinical trials provide an understanding of the patient’s experience of their disease, treatment, and quality of life. Use of PROs in clinical trials is encouraged by both the Food and Drug Administration and European Medicines Agency guidelines for product development.^1^ Hidradenitis suppurativa (HS) is a chronic, recurrent inflammatory condition affecting the apocrine gland-bearing regions of intertriginous skin and is associated with significant psychosocial impacts on patient quality of life.^2^ HS has been reported to affect females at a higher rates than males in North America and Europe.^3^ In HS clinical trials, disease improvement is typically measured using clinician-assessed lesion counts, however, such rating systems do not necessarily correlate with patient quality of life metrics.^4^ PROs are secondary endpoints that can translate how changes in lesion counts meaningfully impact the patient’s life. This is especially worthwhile in HS, in which no cure exists, but strides in pain and quality of life are achievable goals. We performed a systemic review to characterize the utilization of PROs in HS clinical trials and their concordance with trial primary endpoints.

## Methods

**Figure 1.**
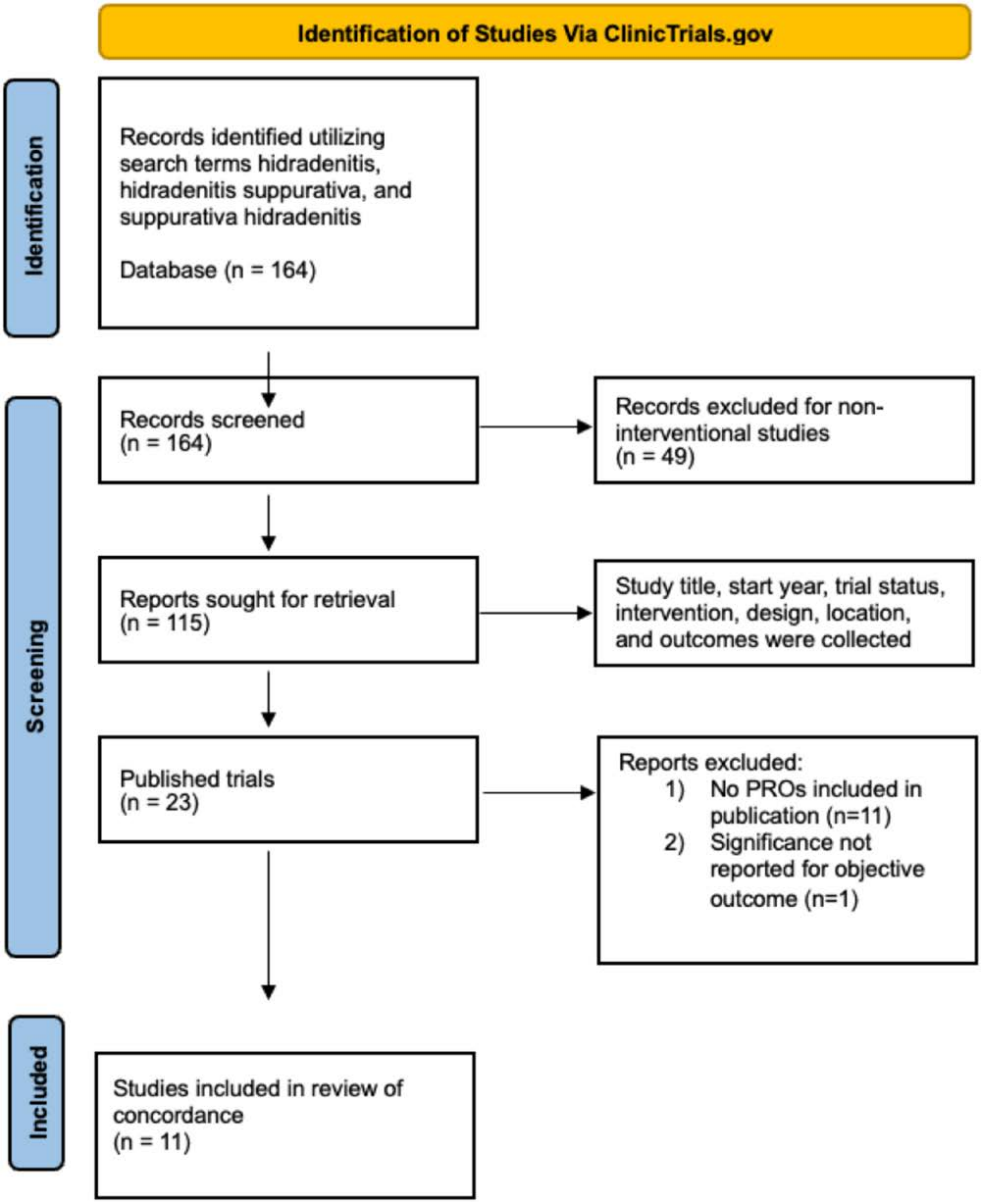
PRISMA 2020 flow diagram for systematic reviews including identification and search terms, records screened and sought for retrieval, excluded trials, total number of published trials, and those studies included in review of concordance. PRISMA, Preferred Reporting Items for Systematic Reviews and Meta-Analyses.

A systematic review of HS clinical trials was performed using the U.S. National Library of Medicine (ClinicalTrials.gov) in June 2023 utilizing the search terms hidradenitis, hidradenitis suppurativa, and suppurativa hidradenitis. Trial phase, location, year, and PRO measures were recorded. To assess the concordance of primary endpoints and PROs, we performed a Preferred Reporting Items for Systematic Reviews and Meta-Analyses 2020 systematic review to identify placebo-controlled trials that included Dermatology Life Quality Index (DLQI) and/or a numeric rating scale (NRS) for pain, the 2 most utilized PROs (Figure 1). Thresholds greater than or equal to 4 DLQI points and greater than or equal to 30% pain NRS score are generally accepted by researchers as minimal clinically meaningful differences for patients to perceive benefit.^5,6^ For each trial and treatment arm, we compared concordance between meaningful improvements in PROs (using aforementioned thresholds) and achievement of the primary endpoint. Outcomes were considered concordantly effective if the primary endpoint was met and PROs showed meaningful improvement compared with placebo. Outcomes were considered concordantly ineffective if the primary endpoint was not met and PROs also did not show meaningful improvement compared to placebo. Few studies reported DLQI as a percentage achieving score of 0 or 1, indicating no impact of disease on quality of life, and this was used to signify meaningful improvement. Significance and standard deviations of PROs were rarely reported, preventing calculation of significance and is a limitation of this review.

## Results

There were 164 HS clinical trials, including 115 interventional studies (Table 1). A total of 47% were Phase II, 14% were Phase III, 12% were Phase I, and 5% were Phase IV among studies with reported trial phase. A total of 65% (*n* = 107) of HS trials included at least one PRO, 42% (*n* = 65) included at least one dermatology-specific PRO, and 16% (*n* = 17) included at least one HS-specific PRO (Table 2). Pain NRS (43%, *n* = 70) and DLQI (42%, *n* = 69) were the most frequently used PRO measures. Use of PROs in clinical trials increased over time, with 4% of trials between 2005 and 2009 utilizing PROs compared to 50% of trials between 2020 and 2023.

**Table 1:** Overview of hidradenitis suppurativa clinical trial characteristics (2005-2023)

Trial characteristics	% (n)
Total HS clinical trials	164
Interventional	70.1% (115)
Noninterventional	29.9% (49)
Trial phase	
Phase 0	0.8% (1)
Phase I	12.2% (14)
Phase I/II	2.6% (3)
Phase II	47.0% (54)
Phase II/III	0.8% (1)
Phase III	13.9% (16)
Phase IV	5.2% (6)
Trial location	
North America	64.0% (105)
U.S	45.7% (75)
Partly U.S.	17.1% (28)
Other	1.2% (2)
Europe	18.3% (30)
Asia	3.7% (6)
International	9.8% (16)
Unknown	4.3% (7)
Trials including at least	
1 PRO	65.2% (107)
Dermatology-specific PRO	64.5% (69)
Disease-specific PRO	15.9% (17)
2 PROs	42.1% (69)
3 PROs	26.2% (43)
Trials with a PRO as primary outcome	14.0% (23)
Trial start years	
2005-2009	4.3% (7)
2010-2014	6.1% (10)
2015-2019	39.6% (65)
2020-2023	50.0% (82)

Overview of all hidradenitis suppurativa clinical trial characteristics from 2005 to 2023 including interventional versus noninterventional study design, trial phase, trial locations, number of patient-reported outcomes, and trial start years.

PRO, patient-reported outcomes.

**Table 2:** PROs in hidradenitis suppurativa clinical trials

Characteristic	No. of trials (%)
Generic PROs	
Pain Numerical Scale	70 (42.7)
Itch Numerical Scale	7 (4.3)
PtGA	8 (4.9)
HADS	4 (2.4)
Treatment satisfaction	4 (2.4)
EQ-5D	4 (2.4)
WPAI	4 (2.4)
SF-36	3 (1.8)
Sleep rating	2 (1.2)
PROMIS	2 (1.2)
POSAS	2 (1.2)
Q-LES	2 (1.2)
WHOQoL	1 (0.6)
FACIT-F	1 (0.6)
Beck depression	1 (0.6)
Dematology-specific PROs	
DLQI	69 (42.1)
Skindex	6 (3.7)
FLQA-d	1 (0.6)
Disease-specific PROs	
HS PGIC	6 (3.7)
HiSQoL	5 (3.0)
HSSDD	4 (2.4)
Odor	4 (2.4)
HSSA	3 (1.8)
HS symptom items	3 (1.8)
HSIA	2 (1.2)
Drainage	2 (1.2)

Tally of specific patient-reported outcomes in all hidradenitis suppurativa clinical trials.

DLQI, Dermatology Life Quality Index; EQ5D, EuroQol-5D, European Quality of Life- 5D Scale; FACTI-F, The Functional Assessment of Chronic Illness Therapy; FLQA-d, Freiburg Life Quality Assessment; HADS, Hospital Anxiety and Depression Scale; HiSQoL, Hidradenitis Suppurative Quality of Life Scale; HS PGIC, Hidradenitis Suppurative Patient Global Impression Scale; HSIA, Hidradenitis Suppurativa Impact Assessment; HSSA, Hidradenitis Suppurativa Symptom Assessment; HSSDD, Hidradenitis Suppurativa Symptom Daily Diary; POSAS, Patient and Observer Scar Assessment Scale; PROMIS, Patient-Reported Outcomes Measurement Information System; PtGA, Patient Global Health Assessment; Q-LES, Quality of Life Enjoyment and Satisfaction Questionnaire; SF-36, Shot Form Health Survey; WHOQoL, World Health Organization Quality of Life; WPAI, Work Productivity and Activity Impairement.

Twelve placebo-controlled HS trials included DLQI and/or pain rating scores.^7–18^ One study did not report significance for provider outcomes and could not be included in the analysis,^9^ leaving 11 total trials or 19 treatment arms (Table 3). Ten of 11 (91%) treatment arms achieving primary endpoint showed concordant clinically meaningful changes in DLQI or pain scores. Two of 8 (25%) treatment arms that failed to achieve the primary endpoint showed concordant insufficient change in PROs. Concordantly effective treatments included adalimumab (40 mg weekly), bimekizumab (320 mg every 2 weeks), povorcitinib (15 mg daily), secukinumab (300 mg every 2 weeks), and infliximab (5 mg monthly).^7,8,10,12,13^ Concordantly ineffective treatments included etanercept (50 mg every 2 weeks) and risankizumab (180 mg at 0, 1, 2, 4, and 12 weeks).^14,15^ Of the 7 discordant treatment arms, 6 showed improvements in PROs but did not achieve primary endpoints: secukinumab (300 mg every 4 weeks), guselkumab (200 mg subcutaneous every 4 weeks and 1200 mg intravenous every 4 weeks), povorcitinib (45 and 75 mg once daily), and risankizumab (360 mg dose at 0, 1, 2, 4, and 12 weeks).^8,10,11,15^ One study of anakinra (100 mg daily) showed significant improvement in lesion counts without minimum meaningful improvements in PROs.^16^

**Table 3:** Concordance of published hidradenitis suppurativa trials

Study	Study design	Study drug	*n*	Study length (weeks)	Change in provider assessed outcome (%)	Statistical significance (y/n)	Clinically meaningful improvement in DLQI (≥4 points or total score of 0 or 1)	Clinically meaningful improvement in Pain (≥30%)	Concordance of at least one PRO
Kirby et al.^8^	Phase 2, placebo-controlled RCT	Povorcitinib 15mg qd	52	16 weeks	48.10%	Yes	35.0% of participants reported DLQI improvement >4 units	44.1% of participants reported a pain reduction ≥30%	Yes (both DLQI and pain)
		Povorcitinib 45mg qd	53	16 weeks	44.20%	No	51.3% of participants reported DLQI improvement >4 units	51.5% of participants reported a pain reduction ≥30%	No
		Povorcitinib 75mg qd	53	16 weeks	45.30%	No	63.2% of participants reported DLQI improvement >4 unit	53.3% of participants reported a pain reduction ≥30%	No
		Placebo	52	16 weeks	28.80%		34.2% of participants reported DLQI improvement >4 units	30.8% of participants reported a pain reduction ≥30%	
Kimball et al.^10^	Phase 3, placebo-controlled RCT	Secukinumab 300 mg q2weeks	181	16 weeks	46%	Yes	48% achieved >4 point change in DLQI	37% reported a pain reduction ≥30% at week 16	Yes (both DLQI and pain)
	Secukinumab 300 mg q4 weeks	180	16 weeks	42%	No	48% achieved >4 point change in DLQI	33% reported a pain reduction ≥30%at week 16	No	
	Placebo	180	16 weeks	30%		29% achieved >4 point change in DLQI	23% reported a pain reduction ≥30% at week 16		
Kimball et al.^11^	Phase 2, placebo-controlled RCT	Guselkumab 200mg sc q4 weeks	59	16 weeks	50.80%	No	8.9% achieved DLQI 0/1	1.6 mean reduction in pain score	No
		Guselkumab 1200 mg IV	60	16 weeks	45%	No	5.0% achieved DLQI 0/1	1.2 mean reduction in pain score	No
		Placebo	62	16 weeks	38.70%		4.8% achieved DLQI 0/1	0.3 mean reduction in pain score	
Kimball et al.^15^	Phase 2, placebo-controlled RCT	Risankizumab 180 mg at 0, 1, 2, 4, and 12 wks	80	16 weeks	46.80%	No	5.1% achieved a final DLQI of 0 or 1	29.2% of patients reported a pain reduction ≥30%	Yes (DLQI and Pain)
		Risankizumab 360 mg at 0, 1, 2, 4, and 12 wks	81	16 weeks	43.40%	No	7.9% achieved a DLQI of 0 or 1	40.0% of patients reported a pain reduction ≥30%	No
		Placebo	82	16 weeks	41.50%		7.4% achieved a DLQI of 0 or 1	33.0% of patients reported a pain reduction ≥30%	
Bechara et al.^17^	Phase 4, placebo-controlled RCT	Adalimumab 160 mg at wk 0, 80 mg wk 2, 40 mg q1w after	103	12 weeks	48%	Yes	13.6 mean improvement	Not reported	Yes (DLQI)
		Placebo	103	12 weeks	34%		12.9 mean improvement		
Glatt et al.^7^	Phase 2, placebo-controlled RCT	Bimekizumab 320 mg q2 weeks	46	12 weeks	57.30%	Yes	36% of participants reported DLQI of ≥1 unit	64% of participants reported a pain reduction ≥30%	Yes (both DLQI and pain)
		Adalimumab 40 mg q1 week	22	12 weeks	60%	Yes	14% of participants reported DLQI of ≥ 1 unit	50% of participants reported a pain reduction ≥30%	Yes (both DLQI and pain)
		Placebo	22	12 weeks	26.10%		0% of participants reported DLQI of ≥ 1 unit	37% of participants reported a pain reduction ≥30%	
Kimball et al.^12^	Phase 3, placebo-controlled RCT	Adalimumab 40 mg q1 week	153	12 weeks	41.80%	Yes	Not reported	27.9% achieved reduction ≥30%	Yes (Pain)
		Placebo	154	12 weeks	26.00%			24.8% achieved reduction ≥30%	
		Adalimumab q1 or 2 weeks	193 weekly, 48 every other week	12 weeks	58.90%	Yes	Not reported	45.7% achieved reduction ≥30%	Yes (Pain)
		Placebo	49	12 weeks	27.60%			20.7% achieved reduction ≥30%	
Tzanetakou et al.^16^	Phase 2, placebo-controlled RCT	Anakinra 100 mg daily	10	12 weeks	78%	Yes	No change (not further reported)	No change (not further reported)	No
		Placebo	10	12 weeks	30%				
Kimball et al.^18^	Phase 2, placebo-controlled RCT	Adalimumab 40 mg q1 week	31	16 weeks	18%	Yes	6.3 units mean DLQI improvement	47.9% of participants reported a pain reduction ≥30%	Yes (Both DLQI and pain)
		Adalimumab 40 mg every other week	38	16 weeks	10%	Yes	3.2 units mean DLQI improvement	36.2% of participants reported a pain reduction ≥30%	Yes (both DLQI and pain)
		Placebo	34	16 weeks	4%		2.3 units mean DLQI improvement	27.1% of participants reported a pain reduction ≥30%	
Adams et al.^14^	Placebo-controlled observational study	Etanercept 50 mg q2 weeks	10	12 weeks	No change in PGA (not further reported)	Not reported	Not reported	No change (not further reported)	Yes (Pain)
		Placebo	10	12 weeks	No change in PGA (not further reported)				
Grant et al.^13^	Phase 2, placebo-controlled RCT	Infliximab 5 mg monthly	18	8 weeks	60% responded with a 25% to less than 50% decrease in HSSI	Yes	10.0 units mean DLQI improvement	Mean pain reduction was 39.8%	Yes (both DLQI and Pain)
		Placebo	15	8 weeks	5.6% responded with a 25% to less than 50% decrease in HSSI		1.6 units mean DLQI improvement	Mean pain reduction was 0.6%	

For the 11 trials that met the criteria, study design, study drug, length, and concordance of provider assessed, and patient-reported outcomes are reported.

DLQI, Dermatology life Quality Index; HSSI, Hidradenitis Suppurativa Severity Index; RCT, randomized control trial.

## Discussion

Our study demonstrates a high concordance (91%) between PROs and the achievement of the primary endpoint in effective trials, and a low concordance (25%) in trials that did not achieve primary endpoint. In studies that did not achieve a primary endpoint, PROs tended to reflect improvement. Effective treatments demonstrating concordance of provider and patient-reported assessments include adalimumab (40 mg weekly), bimekizumab (320 mg every 2 weeks), povorcitinib (15 mg daily), secukinumab (300 mg every 2 weeks), and infliximab (5 mg monthly).^7,8,10,11,13,17,18^ Concordance of provider and patient assessments suggest these to be highly effective medications. Adalimumab, secukinumab, and bimekizumab are already approved for the treatment of HS (bimekizumab is approved in Europe only), while infliximab is widely considered to be a highly efficacious off-label treatment for severe disease. Improvement in PROs makes povorcitinib a promising treatment as it continues down the trial pipeline. Concordantly ineffective treatments, or treatments that failed to achieve primary endpoint and also did not demonstrate clinically meaningful improvements in PROs included etanercept (50 mg every 2 weeks) and risankizumab (180 mg at 0, 1, 2, 4, and 12 weeks).^14,15^ Since PROs often show improvement even when the primary endpoint is not met, the fact that PROs did not show significant improvement suggests that these treatments are unlikely to be effective. Discordant studies included povorcitinib (45 and 75 mg daily), guselkumab (200 mg subcutaneous every 4 weeks and 1200 mg intravenous every 4 weeks), povorcitinib (45 and 75 mg once daily), and risankizumab (360 mg dose at 0, 1, 2, 4, and 12 weeks) and anakinra (100 mg daily).^8,10,11,15,16^ Anakinra was the only treatment that showed significant improvement in lesion counts without improvement in DLQI or pain scores.^16^ Notably, anakinra has not moved forward to larger clinical studies for the treatment of HS. Although the rest did not significantly achieve HiSCR, subjects reported improvement in both DLQI and pain scores. High concordance between provider-assessed and patient-reported outcomes suggests PROs can sensitively identify both effective and ineffective outcomes.

PROs are increasingly being utilized in HS clinical trials. Between 2005 and 2009, 4.3% of studies included PROs. From 2020 to 2023, 65% of trials included at least one PRO and 14% used a PRO as a primary outcome measurement. This reflects the increasing number of clinical trials, including later phase studies that tend to include PROs, as well as the recent emergence of disease-specific PRO measures. DLQI and pain NRS were the most used PRO measures. In 2019, an HS-specific quality of life tool was introduced called the Hidradenitis Suppurativa Quality of Life (HiSQOL) and was recently implemented in the povorcitinib clinical trial.^19^ In the study, HiSQOL paralleled both the provider-graded clinical responses and DLQI, both of which showed respective improvements with increasing doses.^8^

HS is characterized by times of inactivity and flares. While lesion counts provide single snapshots of disease activity, PROs can capture perceptions of emerging or resolving lesions not actively present. In addition, they can clarify disease activity during equivocal physical exams, such as in patients with coalescing abscesses and fistulas, which lead to intergrader variation. While clinical, physical assessments remain necessary primary study endpoints, our study shows that PROs are increasingly being used and correlate with clinical change.

## Conclusion

In 11 published HS trials that included both provider assessments and PROs, 82% of trials contained PROs that concordantly reflected provider-assessed endpoints. Concordance was observed for both successful as well as failed studies. The inclusion of PROs in trials continues to increase with time, with 50% of studies between 2020 and 2023 containing PROs. PROs are a sensitive supportive outcome measure that can provide insights into the patient quality of life over time.

## Conflicts of interest

The authors made the following disclosures: A.R.M. has no relevant disclosures. He has consulted for Kyowa, Eli Lilly, Momenta, UCB, and Regeneron in the past, greater than 24 months ago. He has consulted for PHELEC in the past, great than 12 months. He has consulted for Incyte, Soligenix, Clarivate, and Bristol Myers Squibb in the past, less than 12 months ago. He consults for Argenyx, Boehringer, Janssen, and Ingelheim currently. He consults for Regeneron and Pfizer currently with payments to the institution. He has grant support from Kyowa, Miragen, Regeneron, Corbus, Pfizer, Incyte, Eli Lilly, Aregenx, Palbella, Abbvie, Priovant, Merck in the last 24 months. Beyond 24 months, grant support has come from Sun Pharma, Elorac, Novartis, and Janssen. His current patents include Methods and Materials for assessing and treating cutaneous squamous cell carcinoma (provisional 63-423254), use of oral JAKi in Lichen Planus (provisional 63/453,065), and Topical Ruxolitinib in Lichen Planus (WO2022072814A1). The remaining authors have no conflicts of interest to disclose.

## Funding

None.

## Study approval

N/A

## Author contributions

AG, AH, and SC: Participated in research design, data analysis, and writing of the paper. SA: Participated in data analysis, and writing of the paper. JK and AM: Participated in reviewing and writing of the paper.

## Data availability

Additional data may be acquired by contacting the corresponding author.
